# How Strigolactone Shapes Shoot Architecture

**DOI:** 10.3389/fpls.2022.889045

**Published:** 2022-07-12

**Authors:** Khopeno Khuvung, Federico A. O. Silva Gutierrez, Didier Reinhardt

**Affiliations:** Department of Biology, University of Fribourg, Fribourg, Switzerland

**Keywords:** strigolactone, auxin, cytokinin, abscisic acid, branching, apical dominance, dormancy, BRANCHED1

## Abstract

Despite its central role in the control of plant architecture, strigolactone has been recognized as a phytohormone only 15 years ago. Together with auxin, it regulates shoot branching in response to genetically encoded programs, as well as environmental cues. A central determinant of shoot architecture is apical dominance, i.e., the tendency of the main shoot apex to inhibit the outgrowth of axillary buds. Hence, the execution of apical dominance requires long-distance communication between the shoot apex and all axillary meristems. While the role of strigolactone and auxin in apical dominance appears to be conserved among flowering plants, the mechanisms involved in bud activation may be more divergent, and include not only hormonal pathways but also sugar signaling. Here, we discuss how spatial aspects of SL biosynthesis, transport, and sensing may relate to apical dominance, and we consider the mechanisms acting locally in axillary buds during dormancy and bud activation.

## Introduction

Its central role in the regulation of shoot architecture is arguably the most conspicuous function of the phytohormone strigolactone (SL; Domagalska and Leyser, 2011; Barbier et al., 2019). Indeed, most of the information about SL biosynthesis and SL sensing comes from bushy mutants identified in forward genetic screens in thale cress (*Arabidopsis thaliana*), rice (*Oryza sativa*), petunia (*Petunia hybrida*), and pea (*Pisum sativum*) (reviewed in Al-Babili and Bouwmeester, 2015; Yoneyama and Brewer, 2021). Plant architecture is to a large degree defined by branching patterns, that is the number, position, and size of lateral branches. The extent of branching is controlled by the activity of the main shoot apex, which inhibits the outgrowth of axillary buds along the stem. Axillary meristems are initiated in all leaf axils (Wang et al., 2016b); however, they usually only initiate a few leaf primordia and then become dormant, until they are activated to grow out either in response to endogenous/exogenous developmental signals, or as a consequence of removal (or inactivation) of the main shoot apex. This phenomenon is known as apical dominance (AD; Phillips, 1975).

The central feature of AD is systemic correlative inhibition of bud outgrowth, which is under the control of auxin and SL, involving a mechanism known as auxin canalization (Crawford et al., 2010; Shinohara et al., 2013; Zhang et al., 2020). On the other hand, inducing signals such as cytokinin and sugars are involved in the activation of axillary buds (Domagalska and Leyser, 2011; Rameau et al., 2015; Barbier et al., 2019). While several excellent reviews discuss the function of SL in AD (Domagalska and Leyser, 2011; Rameau et al., 2015; Barbier et al., 2019), we focus here more on spatial aspects of SL biosynthesis and sensing, and on local downstream events in the buds required to inhibit bud outgrowth, and to trigger bud activation, respectively. Furthermore, we discuss parallels in meristem dormancy in annual versus perennial plants.

## Selective Advantage of Branching and Apical Dominance

Plants with just a single shoot meristem suffer extinction if the meristem is damaged. For example, a palm tree infested with the red palm weevil (*Rhynchophorus ferrugineus*) cannot recover after its meristem has been consumed by the larvae (Al-Dosary et al., 2016). This can result in serious damage in infested date palm plantations (El-Sabea et al., 2009). Hence, having extra axillary meristems and multiple branches, as in most dicots, is an important selective advantage. However, shoot branching has to be kept in check to avoid shoot overgrowth and a relative depletion of root biomass (low root:shoot ratio), which would interfere with overall plant fitness. Plants have characteristic root:shoot ratios that are species-specific and genetically determined (Wilson, 1988), but root:shoot ratio can also change in response to environmental factors, such as light, nutrient status, and altitude (Körner and Renhardt, 1987; Shipley and Meziane, 2002).

These considerations highlight the importance of regulation of axillary bud outgrowth. AD contributes to focus the resources of the plant to one (or few) growth points, and eventually, to a limited number of fruits and seeds. Hence, plant fitness and reproductive success are tightly linked with the degree of AD (Aarssen, 1995; Lennartsson et al., 2018). However, the relationship is not simple, since removal of apical buds (experimental or by animal grazing) can either reduce reproductive success, because less fruits can be produced, or it leads to increased reproductive success due to the release of multiple axillary branches with inflorescences, which over-compensate the loss of flowers at the original apex (Aarssen, 1995). These findings raise interesting questions concerning the adaptive mechanisms that may have shaped the evolution of AD and the control of bud outgrowth, in particular in the context of its plasticity towards environmental and developmental factors (e.g., light, mineral nutrients, damage, developmental stage, etc.; Aarssen, 1995). In this context, it is interesting to note that some taxa have integrated the loss-of-apical meristem activity in their developmental programs during the evolution of sympodial branching patterns (Danert, 1958; Schmitz and Theres, 1999; Reinhardt and Kuhlemeier, 2002). Sympodial branching involves the programmed arrest of the apical meristem (often with the production of a terminal flower) and the outgrowth of axillary (lateral) meristems which have a defined life-span before they terminate themselves in a reiterative “stop-and-go” fashion. This sympodial branching pattern is characteristic for the inflorescences of the Solanaceae (Danert, 1958; Schmitz and Theres, 1999; Reinhardt and Kuhlemeier, 2002).

As a general rule, high AD is advantageous in densely populated environments, in which plants compete for nutrients and/or light, whereas harsh conditions (e.g., cold, heat, UV radiation, and strong wind) with scarce vegetation favor bushy shoots with low AD, as for example in alpine environments (Körner, 2003). Considering agricultural crops, strong AD is a favored trait in panicoid cereal crops (e.g., maize and millet), since it tends to increase yield per surface area of cultivated soil, and because simpler shoot architecture facilitates harvest (Doust, 2007). Maize is a prominent example which has been bred from bushy ancestors (the Mexican wild maize teosinte) to plants with a single main shoot axis (Yang et al., 2019). In some high-value vegetable and ornamental crops, e.g., tomato, cucumber, and Chrysanthemum, breeding for desired strong AD has not been achieved yet. Hence, their axillary branches have to be manually pruned (Navarrete and Jeannequin, 2000; Xi et al., 2015; Shen et al., 2019), because they would represent sinks that consume resources and cause yield losses. In contrast, low apical dominance (i.e., high branching) is a favored trait in pooid cereal crops, such as wheat, barley, and oat, in which intense tillering increases yield (Doust, 2007). In addition, crops that were bred for simultaneous fruit ripening, e.g., soybean (Tian et al., 2010) and cotton (McGarry et al., 2016), show decreased indeterminacy of the main shoot, usually associated with increased branching.

**Figure 1:**
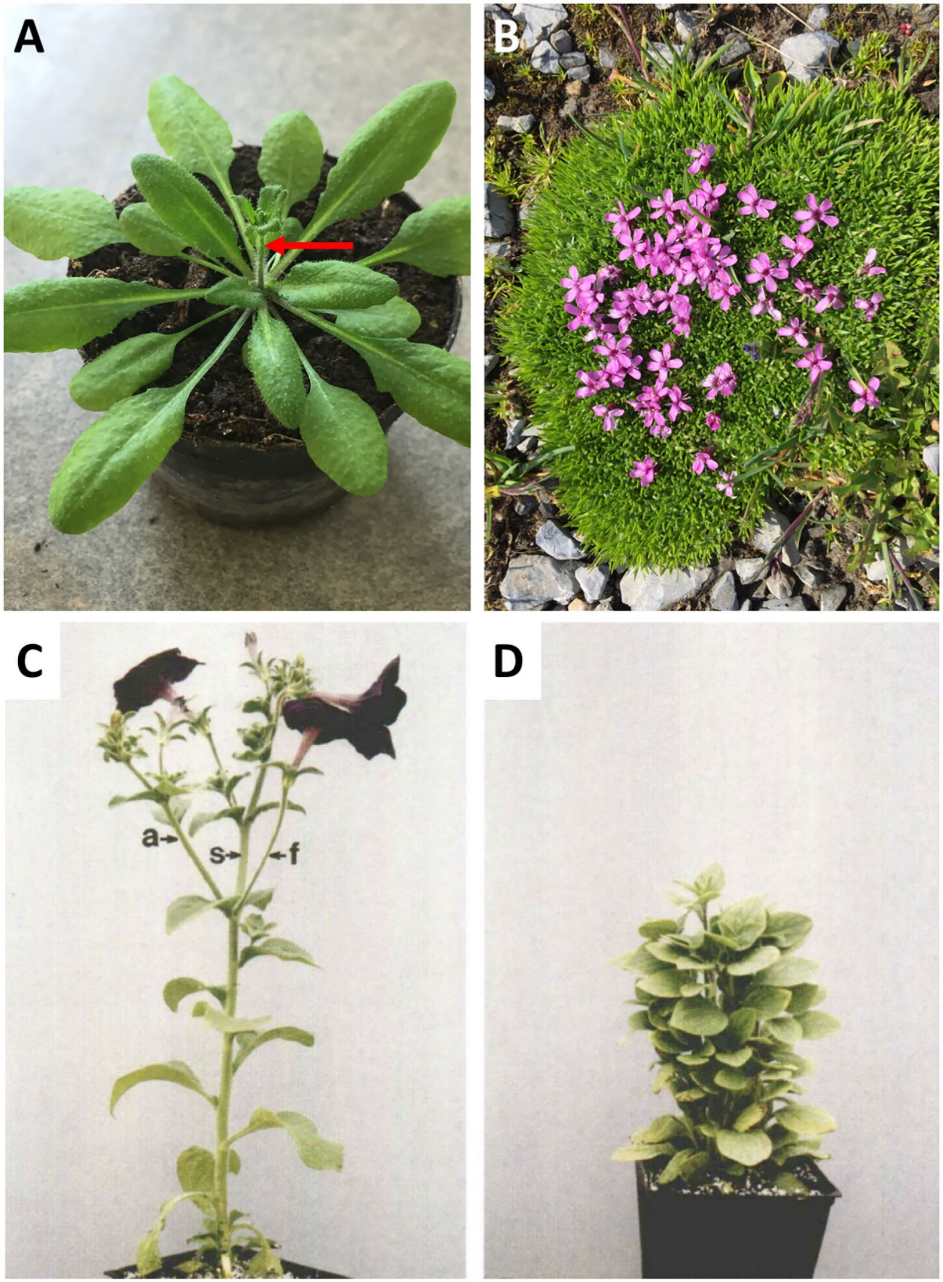
Apical dominance. **(A)** Apical dominance in the pioneer plant *Arabidopsis thaliana* at the onset of flowering. **(B)** The alpine plant *Silene acaulis* exhibits very low apical dominance. It exhibits a profusely branched shoot, in which no main shoot can be distinguished. **(C,D)** The model plant for apical dominance *Petunia hybrida* V26, and the isogenic mutant *decreased apical dominance1* (*dad1*). The pleiotropic phenotype includes short stature, high branching, and late flowering (**C,D**, with permission from Napoli, 1996).

In many plant species, AD is more pronounced during vegetative development, while the onset of flowering coincides with a stimulation of bud outgrowth and increased branching (Hempel and Feldman, 1994; Beveridge et al., 2003; McSteen and Leyser, 2005; Rameau et al., 2015). An example for such a strategy is Arabidopsis, which does not branch during vegetative development, and which initiates a single main inflorescence at the time of bolting (Figure 1A). During the generative phase, several axillary/caulinary branches grow out (Hempel and Feldman, 1994; McSteen and Leyser, 2005), but always much fewer than there are axillary buds. An example of a plant with low AD is the alpine species *Silene acaulis*, which is adapted to harsh climate with strong winds and abundant snow fall (Figure 1B). Mutants with defective AD are highly branched and dwarfed (Beveridge et al., 2003; Snowden and Napoli, 2003; Domagalska and Leyser, 2011; Rameau et al., 2015), in case of petunia to the extent that flowering is delayed (Napoli, 1996; Figures 1C,D), conceivably as a result of resource diversion from the apical inflorescence meristem to the actively growing lateral branches.

## A Central Role for SL in Apical Dominance

A wealth of classical literature documents a central role for polar auxin transport (PAT) in AD and in the regulation of axillary bud outgrowth (Cline, 1991; Leyser, 2005; McSteen and Leyser, 2005). Auxin from apical tissues (in particular young leaves) is transported downward (basipetally) *via* PAT in xylem parenchyma cells, inhibiting bud outgrowth on the way through the stem, however, without entering the buds (Domagalska and Leyser, 2011). A well-founded theory of AD posits that PAT in the stem promotes AD by interfering with auxin canalization and export from the buds (Domagalska and Leyser, 2011). How the lack of auxin canalization is mechanistically related to growth arrest in the bud is not clear, but it may have to do with limited supply of signals and/or resources that would promote meristem activity in the bud. Alternatively, it may impinge on the cell cycle in order to attenuate meristem activity in the bud (Müller and Leyser, 2011). The identification of SL as a second inhibitory element in AD (Gomez-Roldan et al., 2008; Umehara et al., 2008) has raised the question how SL is linked with auxin action. The fact that mutants in either auxin or SL biology have strong defects in AD shows that the effects of the two phytohormones are not redundant. The currently available hypotheses for the action of SL are that it either inhibits auxin canalization from axillary buds to the main stem (by interfering with PAT), or that it directly inhibits bud outgrowth (Domagalska and Leyser, 2011). These hypotheses are not mutually exclusive, and several lines of evidence suggest that they are both valid (see below). Forward genetic screens in Arabidopsis, rice, pea, and petunia for mutants affected in shoot branching have led to the discovery of numerous genes encoding components of SL biosynthesis and signaling (reviewed in Al-Babili and Bouwmeester, 2015; Yoneyama and Brewer, 2021), and missing links in SL biosynthesis and sensing continue to be discovered (Wakabayashi et al., 2021). Taken together, these efforts document the prominent role of SL in apical dominance. The parallel work in these four model species showed how conserved SL biosynthesis and signaling is among flowering plants, and, on the other hand, revealed subtle species-specific differences. Importantly, the parallel approaches allowed to identify signaling elements that are genetically redundant in some of the species, and, therefore, evaded identification in forward mutant screens, as for example the duplicated *MAX2* gene in petunia (Drummond et al., 2012), or the redundant *SMAXL6*, *SMAXL7,* and *SMAXL8* in Arabidopsis (Soundappan et al., 2015). Additional evidence for the role of SL in branching came from crop species such as tomato and potato (Vogel et al., 2010; Pasare et al., 2013). Taken together, these findings substantiate the central and conserved role of SL in the regulation of shoot branching.

## SL Transport Within the Plant: Identifying Sources and Targets of SL by Grafting

**Figure 2:**
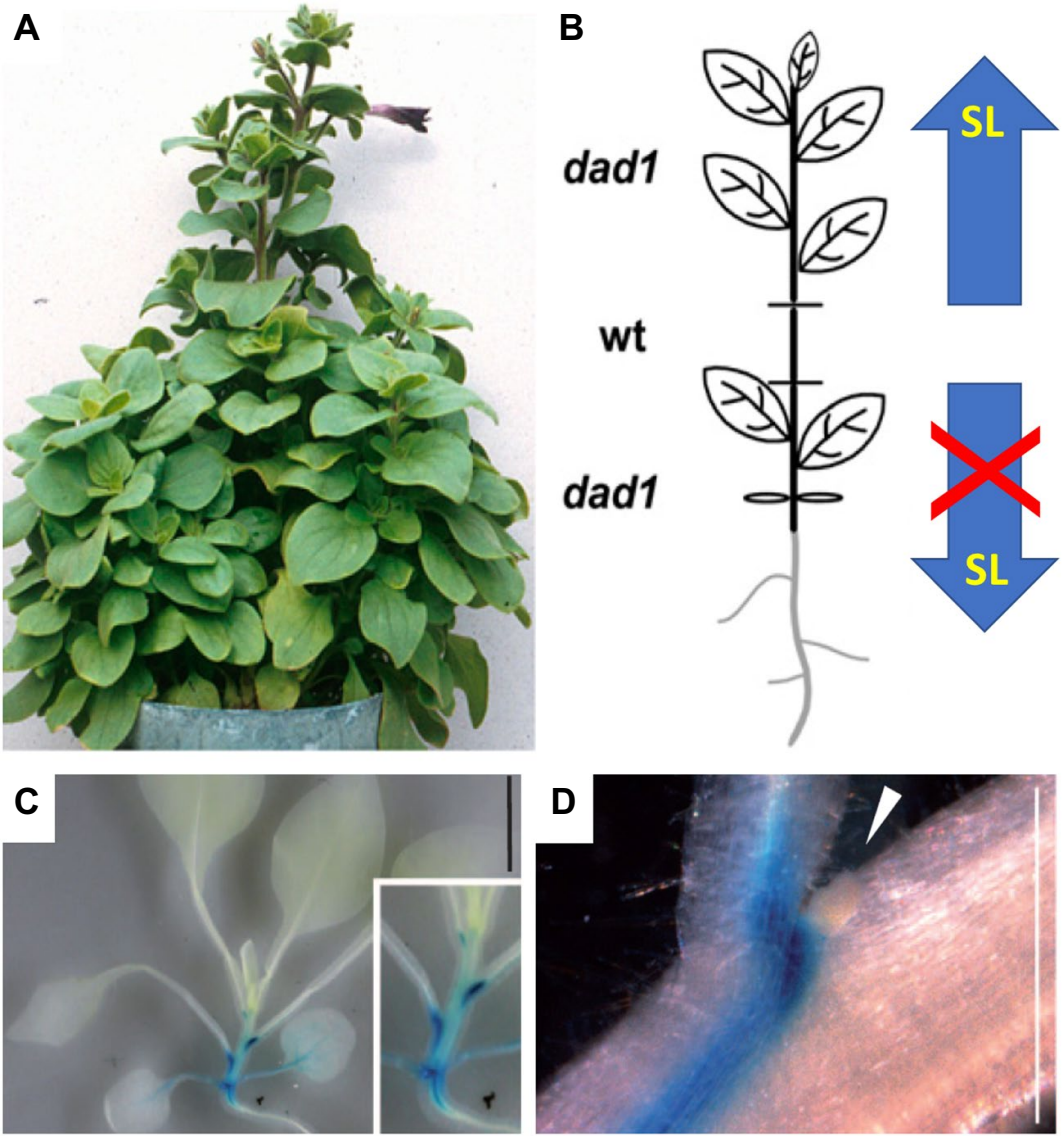
SL transport in the plant. **(A,B)** Grafting experiments have proven acropetal SL transport in the shoot. A small wild-type stem segment (wt) complements the *dad1* phenotype in the scion (top), while the *dad1* stock exhibits the non-complemented high branching phenotype (bottom); thus, only upward SL transport occurred. The scheme in **(B)** represents the organization of the grafted plant in **(A)**. **(C,D)** Expression pattern of the cellular SL transporter *pPhPDR1::GUS* in the shoot of *P. hybrida*. Note highest expression just below the axillary buds, while the buds themselves [arrowhead in **(D)**] show no expression [modified with permission from Simons et al., 2007 **(A,B)** and Kretzschmar et al., 2012 **(C,D)**].

The action of auxin in AD is non-cell autonomous, since it is transported throughout the plant and acts on the buds indirectly (Domagalska and Leyser, 2011). Similarly, SL acts in a systemic fashion and can be transported over long distances in the plant (Mashiguchi et al., 2021). Compelling evidence for spatially separated sites of SL biosynthesis and action comes from grafting experiments with mutants that are defective in SL biosynthesis or sensing (Figure 2). Shoot-to-root grafting in Arabidopsis, petunia, and pea revealed that a wild-type root stock can establish normal AD in an SL-deficient mutant scion, indicative of acropetal SL transport from the root to the shoot (Beveridge, 2000; Booker et al., 2005; Dun et al., 2009; Waldie et al., 2014). Even a relatively small inter-graft between a mutant stock and a mutant scion was sufficient to restore AD to the mutant scion (Napoli, 1996; Simons et al., 2007; Hepworth, 2012) but not to the mutant stock, showing that SL transport is strictly unidirectional (Figures 2A,B; Foo et al., 2001; Simons et al., 2007). Although SL can be transported over long distances (from the root to the shoot), it is not clear whether this transport is required for AD. Wild-type scions grafted on SL-defective mutant stocks grow normally, showing that for AD, SL production in the shoot can be sufficient, at least in such grafts, implying that SL transport from the root may not be necessary for normal AD.

The fact that SL can be transported acropetally raises the question concerning the transport route. Root-to-shoot transport could proceed by mass flow with the transpiration stream in the xylem, or by cellular transport, as in the case of PAT (Petrasek and Friml, 2009; but in the reverse direction). Support for a xylem route of SL transport came from the detection of SL in xylem sap of Arabidopsis and tomato (Kohlen et al., 2011); however, these findings were not confirmed by subsequent work on various plant species, including Arabidopsis and tomato (Xie et al., 2015; see also below).

An alternative route of SL translocation is cell-to-cell transport in a way analogous to PAT (Borghi et al., 2016). Indeed, the first cellular SL transporter identified in *P. hybrida* (PDR1), an ABC transporter of the G-type subfamily, was shown to functionally contribute both to mycorrhizal symbiosis (by targeted secretion from the root), and to AD in the shoot (Kretzschmar et al., 2012). *PDR1* is expressed in root and stem tissues (Kretzschmar et al., 2012), with highest levels at the nodes, next to the axillary buds (Figures 2C,D). PDR1 protein is localized to the plasma membrane, and, based on its expression pattern and loss-of-function mutant phenotype, is likely to function as an SL exporter (Kretzschmar et al., 2012). *Pdr1* mutants exhibit premature bud outgrowth (Kretzschmar et al., 2012), indicating that SL transport to the buds contributes significantly to AD. However, long-distance transport of SL appears to be independent of PDR1 (Shiratake et al., 2019); thus, the mechanism of SL translocation from the root to the shoot remains unclear (Wheeldon and Bennett, 2021).

## Spatial Regulation of SL-Biosynthetic Genes

Powerful tools to identify the sites of action of genes are promoter::reporter constructs (Jefferson et al., 1987; Chalfie et al., 1994) that show gene expression patterns with great spatial resolution. While fluorescent proteins are often the marker of choice because they allow identification in undisturbed live tissues with cellular resolution, they have the disadvantage that the optical permeability of live plant tissues is often limited, and, in addition, autofluorescence of many plant components (cell walls, secondary metabolites in vacuoles, etc.) considerably hampers their analysis. A widely used alternative is the use of enzymatic reporters such as the beta-glucuronidase gene (UidA), also known as the GUS gene, which generates (from the substrate X-gluc) a blue insoluble deposit (5,5′-dibrom-4,4′-dichlor-indigo), which is stable enough to allow for complete tissue clearing and embedding in paraffin or resin for sectioning. Importantly, cleared plant tissues have no blue background color, thus eliminating problems with endogenous background staining (Figures 2C,D).

**Figure 3:**
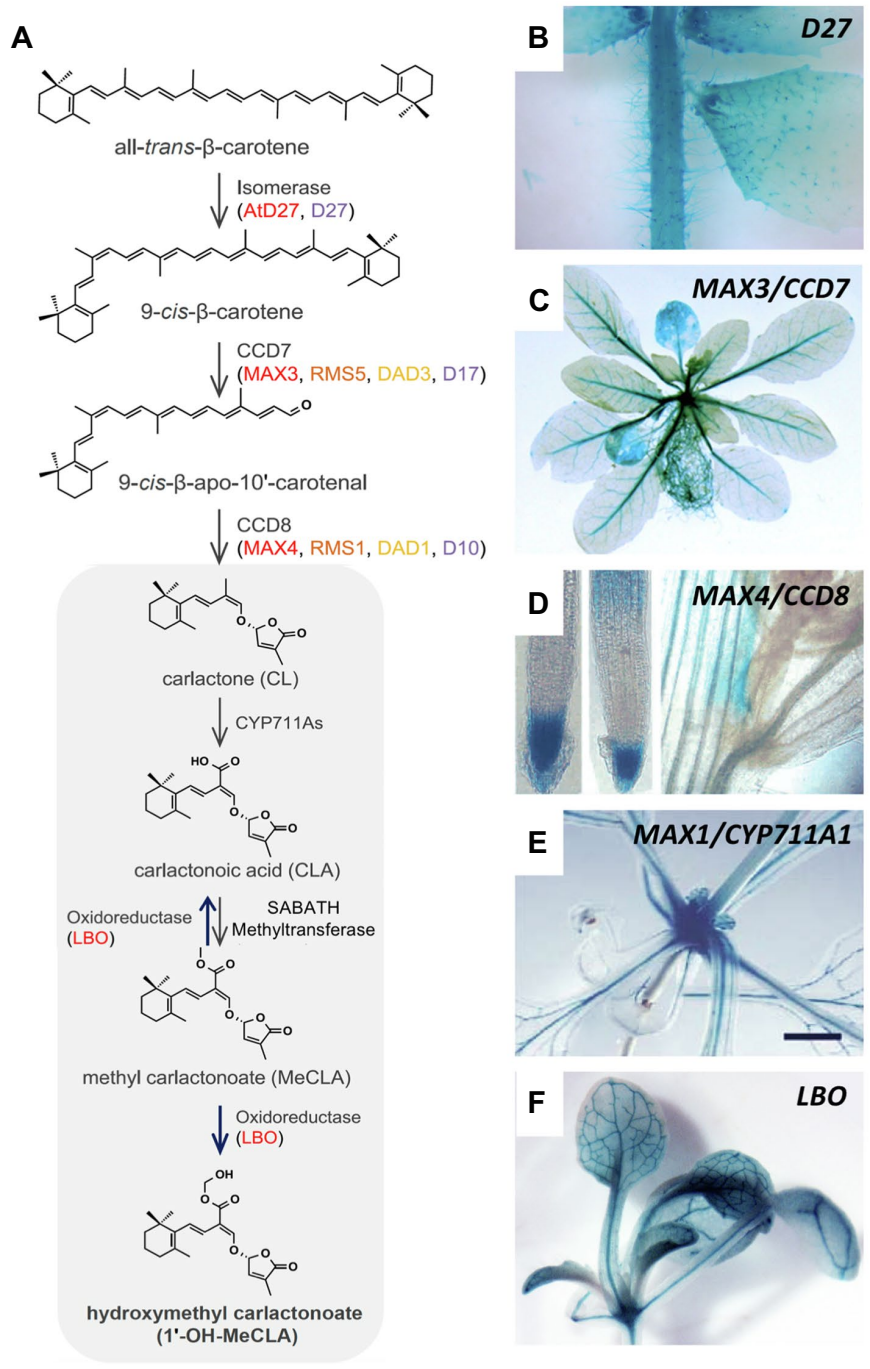
Expression pattern of SL-biosynthetic genes in Arabidopsis. **(A)** Biosynthetic pathway of strigolactone comprising the activity of the isomerase D27, the carotenoid cleavage dioxygenases CCD7 and CCD8, CYP711A, the oxidoreductase LBO, and the SABATH methyltransferase. **(B–F)** Expression pattern of Arabidopsis SL-biosynthetic genes such as *D27* **(B)**, *CCD7*/*MAX3* **(C)**, *CCD8*/*MAX4* **(D)**, *CYP711A1*/*MAX1* **(E)**, and *LBO* **(F)**, as indicated, revealed by promoter::GUS analysis. Note prominent expression in the vasculature of *MAX3*, *MAX1*, and *LBO* [**C,E,F**; modified with permission from Yoneyama and Brewer, 2021 **(A)**; Abuauf et al., 2018 **(B)**; Liang et al., 2011 **(C)**; Sorefan et al., 2003 **(D)**; Booker et al., 2005 **(E)**, and Brewer et al., 2016 **(F)**].

Promoter::GUS analysis with the major SL-biosynthetic genes in Arabidopsis revealed that several of them are active in the vasculature (besides other sites of expression), and some of them (*MAX3*, *MAX1*; *LBO*) are expressed almost exclusively along vascular strands (Figure 3; Booker et al., 2005; Liang et al., 2011; Brewer et al., 2016). The expression of SL-biosynthetic genes along the vasculature was also found in rice for D27 (Lin et al., 2009) and CCD7 (Zou et al., 2006). Hence, it can be assumed that SL, or an SL precursor such as carlactone (Alder et al., 2012), is produced along the vascular system. Although all SL-biosynthetic genes are expressed mainly in the root, they also show expression in aerials tissues, in particular the stem, in Arabidopsis (Figure 3), as well as in other species (Zou et al., 2006; Drummond et al., 2009; Dun et al., 2009; Lin et al., 2009; Vogel et al., 2010; Pasare et al., 2013). This provides a plausible explanation for the fact that in Arabidopsis, petunia, and pea, wild-type scions grafted onto SL-defective mutant stocks are self-sufficient for SL production (Beveridge et al., 1994, 1996, 1997; Napoli, 1996; Morris et al., 2001; Turnbull et al., 2002; Sorefan et al., 2003; Booker et al., 2005; Simons et al., 2007; Drummond et al., 2009).

The vicinity of SL-biosynthetic gene expression to the xylem strands could explain why SL can be detected in the transpiration stream (Kohlen et al., 2011). SL produced along the vasculature could be loaded to the xylem by cellular transporters or by diffusive release from biosynthetic cells. It would then be continuously translocated to the shoot with the transpiration stream, even at low concentrations. While acropetal SL transport may not be essential for AD (see grafting experiments discussed above), SL transport through the xylem could represent a significant contribution to SL function in other aspects of shoot development, e.g., for the regulation of leaf senescence (Ueda and Kusaba, 2015).

## Nutritional Control of Shoot Branching Impinges on the SL Pathway

In addition to the above-mentioned factors that influence AD, nutrients influence shoot architecture, since well-fertilized plants tend to branch more than nutritionally starved plants (Cline, 1991; Czarnecki et al., 2013; Wang et al., 2019; Hou et al., 2021). This effect can be explained, at least partially, by the fact that nutrients, in particular nitrogen (N) and phosphorus (P), impinge on the auxin- and SL-related mechanisms involved in AD (Sakakibara et al., 2006; Yoneyama and Brewer, 2021; Marro et al., 2022). High N status not only promotes branching, an effect that requires auxin and SL signaling (De Jong et al., 2014), but also involves the activation of cytokinin biosynthesis (Takei et al., 2002; Sakakibara et al., 2006; Xu et al., 2015). High P status represses SL-biosynthetic genes, consistent with the observation that SL secretion from the root system is repressed by P fertilization (Yoneyama et al., 2007a,b; Kohlen et al., 2011). P-replenished plants exhibit increased branching (Czarnecki et al., 2013; Wang et al., 2019), conceivably as a result of reduced SL biosynthesis (Umehara et al., 2010; Abuauf et al., 2018; Yoneyama and Brewer, 2021). In P-starved plants, SL biosynthesis is induced (Yoneyama and Brewer, 2021), presumably resulting in acropetal SL transport into the buds. Under these conditions, xylem transport of SL could become relevant (Kohlen et al., 2011), in particular since SL-biosynthetic genes are expressed along the vasculature (Figure 3). However, the role of the xylem in acropetal SL transport is a matter of debate (Xie et al., 2015), and the broad expression pattern of SL-biosynthetic genes throughout the shoot suggests that acropetal SL transport may not be necessary for apical dominance (see above).

## Sites of SL Sensing and Consequences for AD

**Figure 4:**
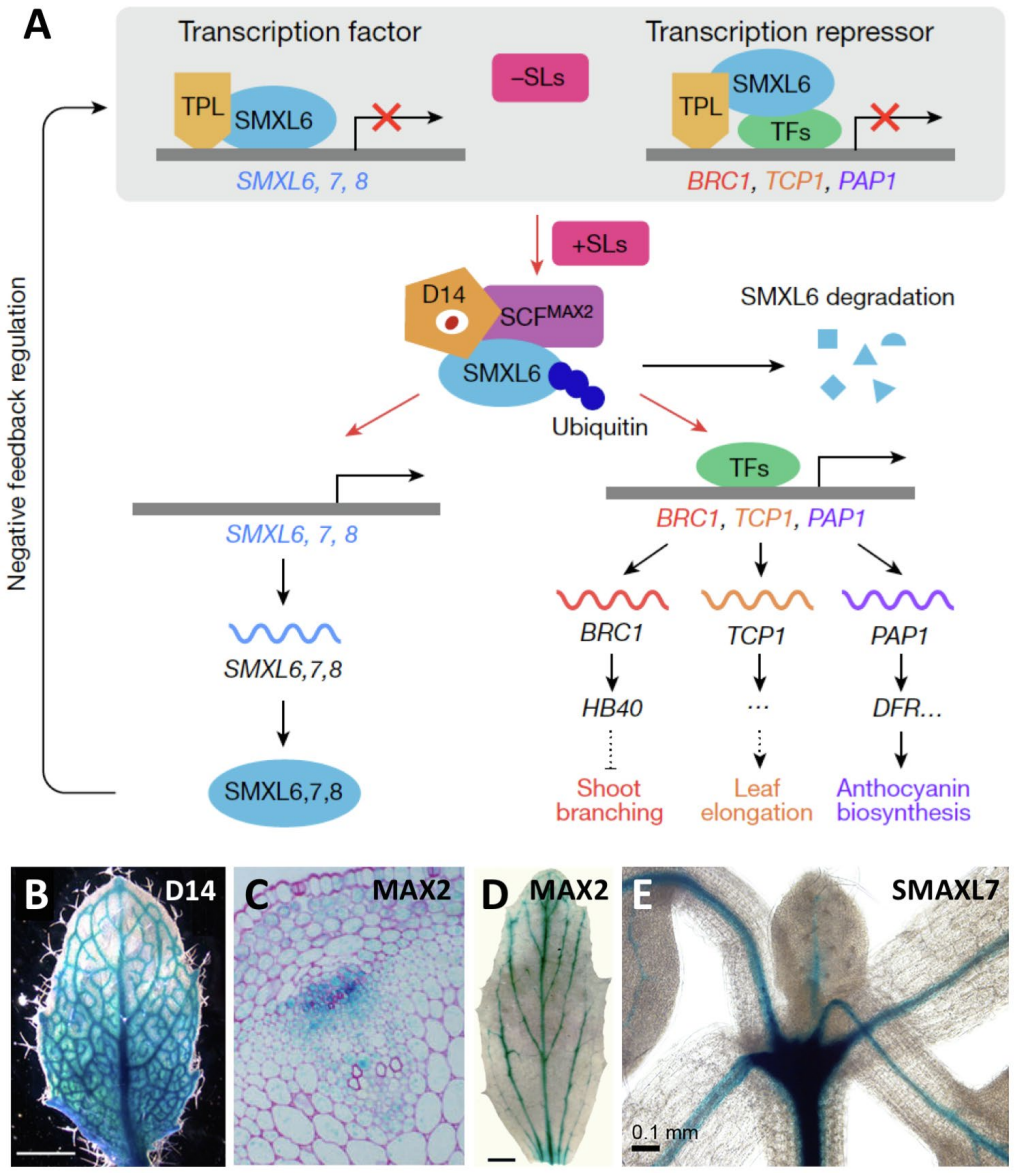
Expression pattern of SL-sensing genes in Arabidopsis. **(A)** Schematic representation of the elements involved in SL perception and signal transduction, including the SL receptor D14, the ubiquitin conjugating enzyme complex SCF^MAX2^ containing the F-box protein MAX2, and the redundantly acting transcriptional repressors SMAXL6, SMAXL7, and SMAXL8, which regulate the expression of target genes such as *BRC1*. **(B–E)** Expression pattern of the SL perception and signaling genes *D14* **(B)**, *MAX2* **(C,D)**, and *D53/SMAXL7* **(E)**, as indicated, revealed by promoter::GUS analysis. Note highest expression in vascular strands as revealed in whole-mount preparations **(B,D,E)**, and in a transverse section of the stem [**C**; modified with permission from Wang et al., 2020 **(A)**; Chevalier et al., 2014 **(B)**; Stirnberg et al., 2007 **(C)**; Shen et al., 2007 **(D)**; and Soundappan et al., 2015 **(E)**].

Interestingly, several SL-sensing genes (*D14*, *MAX2*, *SMAXL6*, *SMAXL7*, and *SMAXL8*) share the expression pattern along the vascular strands with SL-biosynthetic genes (Figure 4; Gao et al., 2004; Shen et al., 2007; Stirnberg et al., 2007; Chevalier et al., 2014; Soundappan et al., 2015; Song et al., 2022). In general, the identity of the cells that express SL-sensing genes along the vasculature is uncertain; however, for the SL receptor D14, expression was attributed to the phloem in Arabidopsis roots (Chevalier et al., 2014), and in the axillary buds of rice (Kameoka et al., 2016), suggesting that SL perception may be possible in these tissues. An association of SL-sensing genes with the vasculature is striking given the function of SL as inhibitor of PAT, which is located to the xylem parenchyma cells (Petrasek and Friml, 2009). Hence, SL perception in these cells would allow for a direct regulation of PAT in these cells. It will be important to identify the sites of SL sensing in more detail, with refined promoter::reporter studies, and with complementation experiments, in which SL-sensing genes are expressed in the respective mutant background under the control of cell-specific promoters. Further insight into SL sensing will come from fluorescent SL reporters, which allow to identify sites of high SL levels in living plant tissues with cellular resolution (Song et al., 2022).

**Figure 5:**
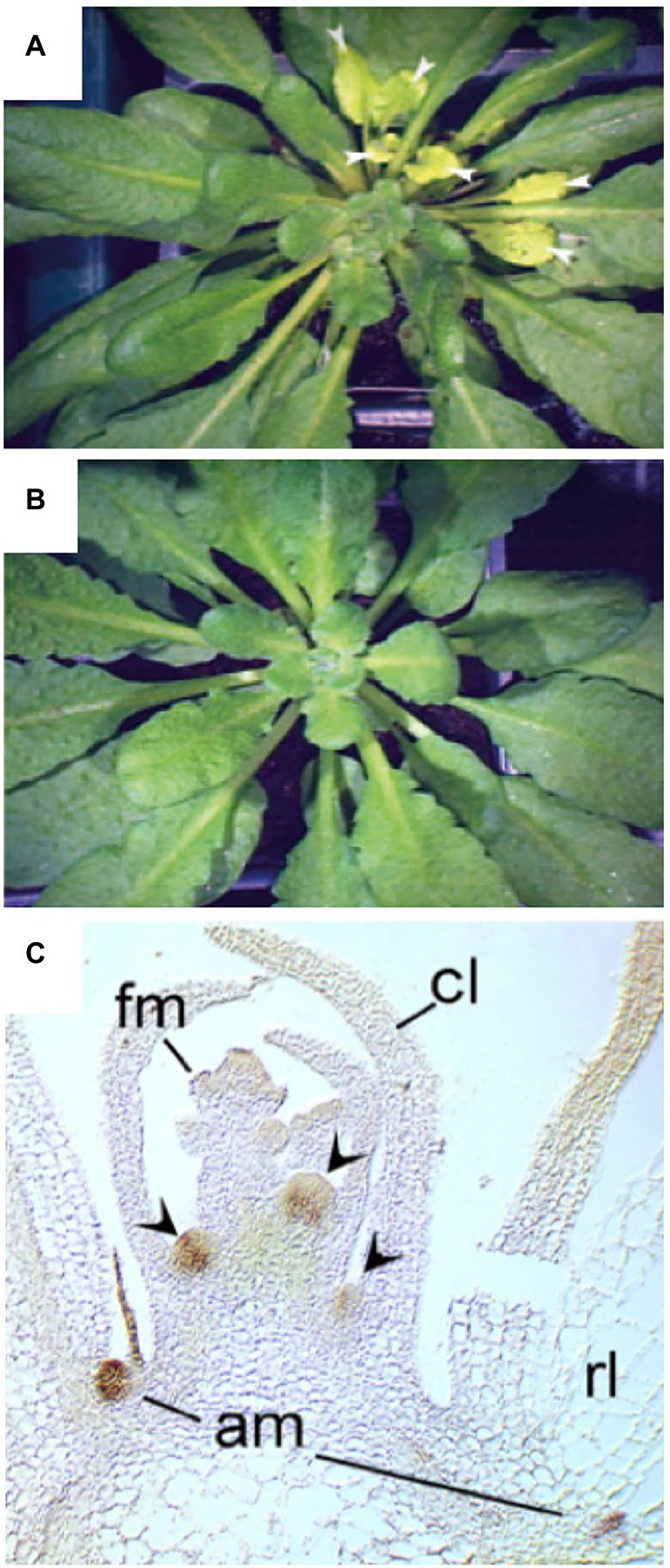
Local action of SL through *MAX2* and *BRC1* in leaf axillary meristems. **(A,B)** Clonal analysis with *max2* mutant sectors reveals local function of *MAX2* in axillary buds. A sector marked by chlorophyll deficiency (yellowish leaves) comprised a single axillary bud that grew out to produce numerous leaves (arrowheads), that are identified as a *max2* mutant sector by the cosegregating genetic defect in chlorophyll biosynthesis **(A)**. A wild-type control plant of the same age does not show bud outgrowth, nor yellowish leaves **(B)**. **(C)** Expression analysis of *BRC1* by *in situ* hybridization reveals expression in axillary meristems (am), but not in floral meristems (fm) at the shoot apex, cl, cauline leaf; rl, rosette leaf. Modified with permission from Stirnberg et al., 2007 **(A,B)**, and Aguilar-Martinez et al., 2007 **(C)**.

A powerful tool to assign biological function to precisely defined cell populations is clonal analysis, in which the fate of genetically distinct cell lineages is followed in chimeras (Buckingham and Meilhac, 2011), a technique that has been pioneered in plants (Poethig, 1987). Clonal analysis has shown that SL perception in axillary buds acts locally (Stirnberg et al., 2007). Introduction of a mutation in the *MAX2* gene encoding the F-BOX protein component of the SL-sensing machinery, comprising a sector with a single axillary bud, is sufficient to release its meristem from AD (Figures 5A,B). The fact that the surrounding wild-type tissues were not able to functionally complement the *max2* defect in the axillary bud (Stirnberg et al., 2007) shows that SL perception acts locally to inhibit bud outgrowth. However, SL perception and sensing may not be entirely cell autonomous, since D14 was shown to be mobile over several cell diameters from the meristem base to the stem cells of axillary buds in rice (Kameoka et al., 2016), and was even graft-transmissible in pea (Beveridge et al., 1996). Furthermore, D14 protein was detected in phloem sap by proteomic analysis (Aki et al., 2008; Batailler et al., 2012), and by microscopic analysis of GFP-tagged D14 protein (Kameoka et al., 2016). Taken together, these results indicate that D14 protein is transported from cell to cell and by phloem transport (Chevalier et al., 2014; Kameoka et al., 2016; Barbier et al., 2019). This may allow D14 to function in the meristem proper of the axillary buds, in which the *D14* promoter is not expressed (Kameoka et al., 2016).

## Which Pathways Act Downstream of SL to Prevent Bud Outgrowth?

While it is clear that auxin and SL impose dormancy on axillary buds, it is less clear how exactly growth and organogenesis is inhibited in the axillary meristems. Is the cell cycle attenuated? Are the meristems metabolically starved? Or is there an additional inhibitory principle involved? The auxin canalization model, including SL as a major player, can explain many aspects of correlative inhibition between the shoot tip and axillary buds, but how does it interfere––locally––with growth of the buds? On the other hand, the direct signaling model posits that SL prevents axillary growth through the inhibitory transcription factor BRANCHED1 (BRC1) in Arabidopsis (Aguilar-Martinez et al., 2007). BRC1 is functionally conserved in monocots and dicots (Aguilar-Martinez et al., 2007; Brewer et al., 2009; Finlayson et al., 2010). Interestingly, gain-of-function alleles of the *BRC1* orthologue in maize, *Teosinte branched1* (*Tb1*), have been selected for during domestication of modern maize for low branching (Dong et al., 2019a).

*BRC1* is expressed at high levels in dormant buds (Figure 5C), and *brc1* mutants exhibit excessive branching, consistent with a role of *BRC1* in AD (Aguilar-Martinez et al., 2007). SL can activate *BRC1* expression (Wang et al., 2019, 2020; Kerr et al., 2020, 2021), indicating that SL could act directly in the buds to inhibit bud outgrowth. Although BRC1 is probably not the only inhibitor of bud outgrowth (Seale et al., 2017), it is a conserved central player in Arabidopsis, pea, tomato, and maize (Martin-Trillo et al., 2011; Braun et al., 2012). Interestingly, TB1 homologues in cereals have functionally diversified to control ear architecture in crop-specific ways (Dong et al., 2019a). This is likely to reflect the particular development of reproductive structures in cereals (ears, tassels; Dong et al., 2019a), which represent highly branched generative shoot axes (Wang et al., 2021).

As a TCP-type transcription factor, BRC1 can be expected to act through activation (or repression) of downstream genes, which could provide a hint regarding the action mechanisms in AD. *BRC1* itself is under transcriptional control by SL through the action of the transcription factors SMAXL6, SMAXL7, and SMAXL8, which promote bud outgrowth through inhibition of *BRC1* expression (Figure 4A; Soundappan et al., 2015; Wang et al., 2020). BRC1 directly activates several homeobox proteins to mediate bud dormancy in Arabidopsis (Gonzalez-Grandio et al., 2017), and an orthologous transcriptional mechanism involving TB1 and GT1 controls branching in maize (Dong et al., 2019a). Hence, genetic evidence indicates that the molecular mechanism controlling bud dormancy may be conserved between monocots and dicots. How does the BRC1/TB1 nexus regulate branching? RNAseq and CHIPseq analysis in Arabidopsis showed that BRC1, in concert with several homeobox proteins, activates abscisic acid (ABA) biosynthesis by NCED3 in axillary buds (Gonzalez-Grandio et al., 2017). Similarly, TB1 acts through ABA to inhibit axillary buds in maize (Dong et al., 2019b). This includes activation of ABA biosynthetic genes in axillary buds during dormancy (Luo et al., 2019).

Interestingly, bud dormancy during the resting period (e.g., winter) in perennial plants such as poplar also involves ABA (Pan et al., 2021). The finding that BRC1 acts through ABA in axillary buds may explain the overlap between *max2* phenotypes and ABA signaling in drought resistance (Bu et al., 2014) and in the resistance against bacterial pathogens (Piisilä et al., 2015). On the other hand, it is consistent with reports that have shown a role for ABA in the inhibition of axillary branching in Arabidopsis and maize (Cline and Oh, 2006; Yao and Finlayson, 2015; Cao et al., 2020). One might ask why then no ABA-related mutants were identified in screens for increased branching? ABA has numerous roles in plant development from seed dormancy to regulation of leaf transpiration and stress responses; therefore, ABA-deficient and ABA-insensitive mutants have rather pleiotropic phenotypes (Nambara and Marion-Poll, 2005; Cutler et al., 2010), which could potentially mask quantitative branching phenotypes. Nevertheless, ABA biosynthetic mutants such as *nced3* and *aba2* showed branching phenotypes in the context of phytochrome-dependent regulation of shoot branching (Reddy et al., 2013), a phenomenon that involves the canonical BRC1-dependent pathway (Gonzalez-Grandio et al., 2013).

## How Are Buds Triggered to Grow Out When They Are Released From Dormancy?

Given the fact that bud dormancy is mediated by auxin and SL, it could be assumed that the activation of bud outgrowth (in response to environmental cues or after decapitation) may require simply the release from this inhibitory mechanism. Indeed, the highly branched mutant phenotypes of auxin-insensitive (Stirnberg et al., 1999) and SL-deficient (Beveridge et al., 2003; Domagalska and Leyser, 2011; Rameau et al., 2015) mutants show that the inactivation of auxin- and SL-mediated AD is sufficient to promote bud outgrowth. However, does this also apply to the rapid events triggered by decapitation? Several lines of evidence suggest that activation of dormant buds involves additional mechanisms independent of auxin and SL.

Cytokinin has long been known to promote growth of axillary branches in various plant species (Sachs and Thimann, 1967; Chatfield et al., 2000; Tanaka et al., 2006; Ferguson and Beveridge, 2009; Dun et al., 2012; Chen et al., 2013; Young et al., 2014), suggesting that it may contribute to bud activation following decapitation (Shimizu-Sato et al., 2009), or in response to favorable light conditions (Roman et al., 2016). Cytokinin biosynthesis is inhibited by auxin (Tanaka et al., 2006), while SL induces a CK-degrading oxidase (Duan et al., 2019), conversely, decapitation leads to the induction of CK biosynthetic genes and increased CK levels in the vicinity of the buds (Tanaka et al., 2006), consistent with a role of CK in bud activation (Shimizu-Sato et al., 2009; Müller et al., 2015).

However, CK may not be the first, and not the only element in bud activation. In pea, one of the first signs of bud activation can be observed after just a few hours from decapitation, long before changes in auxin transport and canalization can be expected to result in the release of buds, and before CK can have accumulated to induce bud outgrowth (Mason et al., 2014). This argues for the involvement of a rapid activating principle in bud activation. This signal has been assigned to sucrose and trehalose-6-phosphate (T6P), whose levels increase rapidly after decapitation (Fichtner et al., 2017, 2021; Barbier et al., 2019). In agreement with such a scenario, elegant recent work with cell-specific genetic manipulation of phloem transport (Paterlini et al., 2021) and sugar supply (Fichtner et al., 2021) indicated that sugars may indeed contribute to axillary bud activation in Arabidopsis. Since *BRC1* expression is repressed by sugars (Mason et al., 2014; Barbier et al., 2015; Otori et al., 2019; Patil et al., 2022), a plausible model is that sugars contribute to bud outgrowth by attenuating BRC1-dependent dormancy (Wang et al., 2019). The role of sugars in bud activation is likely to represent a signaling function, since non-metabolizable sugars can mimic the effects of sucrose and T6P (Rabot et al., 2012; Barbier et al., 2015, 2021).

A connection between sugar activation and SL signaling has been revealed in rice, where sucrose interferes with SL signaling by repressing a component in SL perception (D3) and destabilizing the SL receptor D14, whereas their target D53, a promoter of bud outgrowth, is stabilized by sucrose, ultimately resulting in reduced expression of BRC1/TB1 (Patil et al., 2022). Similar effects were found in pea (Bertheloot et al., 2020; Patil et al., 2022), suggesting that the antagonistic action of sugars against SL signaling may be conserved in angiosperms. Ultimately, the release from BRC1/TB1, together with the induction of cytokinin levels (Müller and Leyser, 2011), results in the activation of the cell cycle and of basic cell metabolism (incl. protein synthesis and primary metabolism; Devitt and Stafstrom, 1995; Gonzalez-Grandio et al., 2013; Luo et al., 2019; Dong et al., 2019b), which are required to promote outgrowth and organogenesis in the axillary meristems (Müller and Leyser, 2011).

**Figure 6:**
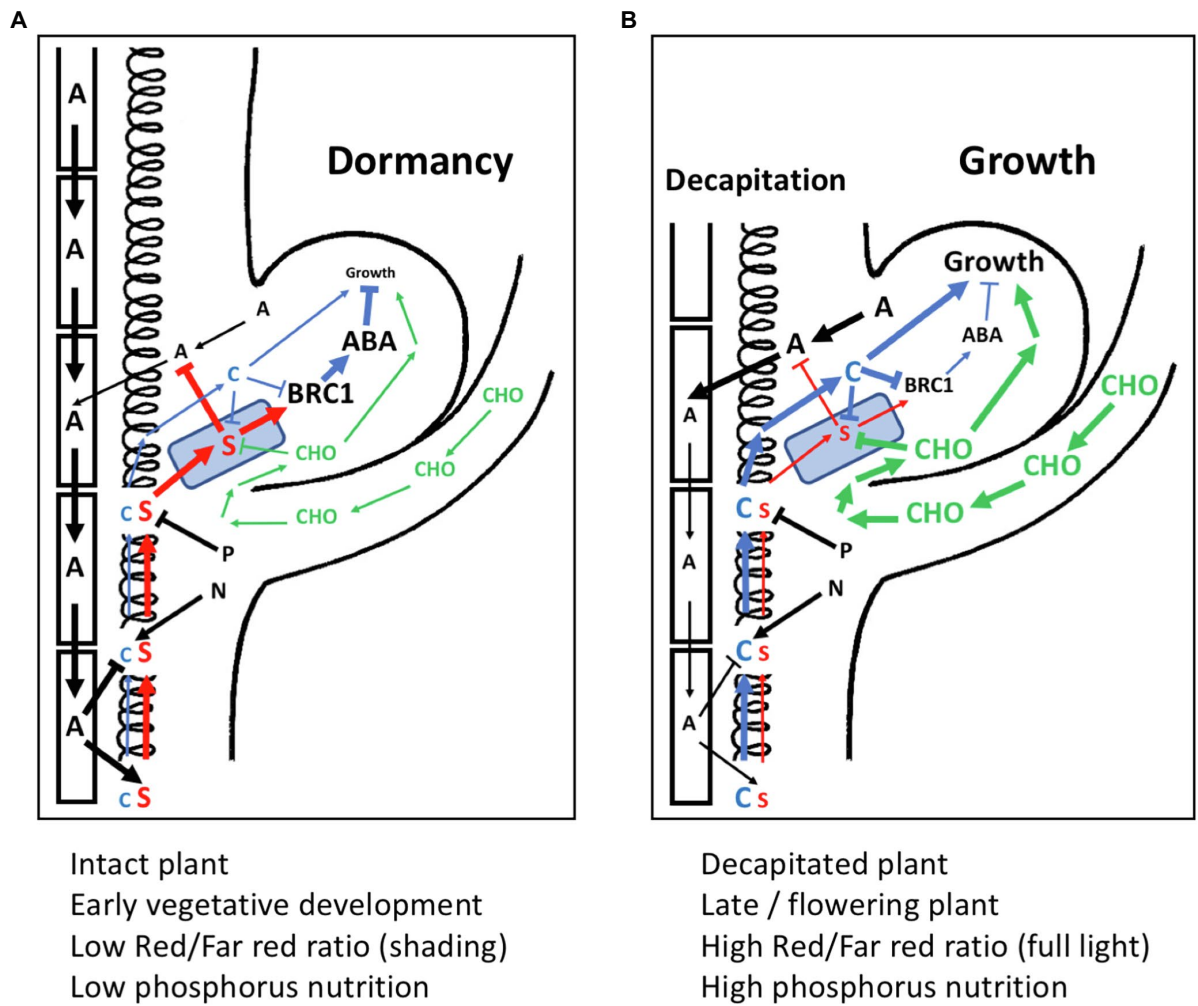
Model for the interactions of SL with other components in apical dominance. **(A)** An axillary bud under the influence of apical dominance with active polar auxin transport stream in the stem, which inhibits cytokinin (C) biosynthesis, and stimulates strigolactone (S) production. Strigolactone enters the buds *via* PDR1 transport (represented by a blue PDR1-expressing square). Strigolactone inhibits auxin canalization from the bud, and stimulates *BRC1* gene expression. BRC1 activates the ABA dormancy program, resulting in growth arrest. The stimulatory effect of nitrogen (N) on cytokinin biosynthesis and the inhibition of strigolactone biosynthetic genes by phosphorus (P) are indicated, although they are at a steady-state intermediate level and do not influence bud activity. Relative signaling strength is represented by font size and thickness of the arrows. **(B)** Situation as in **(A)** depicting changes upon decapitation of the main shoot apex. Sugars (CHO) rapidly enter the bud, where they interfere with strigolactone sensing, and rapidly stimulate growth. Polar auxin transport in the stem is weak, reducing strigolactone biosynthesis and releasing the inhibition of cytokinin biosynthesis in the stem. Lower strigolactone levels allow canalization of auxin from the bud, while increased cytokinin levels further stimulate bud outgrowth by reducing BRC expression. Relative signaling strength is represented by font size and thickness of the arrows.

Is there a conflict between the models of bud inhibition (auxin canalization vs. direct SL-dependent inhibition), or between the mechanisms assumed to mediate bud activation (onset of local auxin canalization in the bud vs. sugar activation)? These alternative mechanisms are not necessarily mutually exclusive. The collective evidence shows that SL can promote AD both, locally in the buds (through BRC1), and systemically, by modulating auxin canalization (Figure 6). Similarly, bud activation could independently involve both sugar activation and the onset of auxin canalization and cytokinin accumulation in the bud (Figure 6). The relative importance, and the dynamics, of these processes may differ between plant species, to the extent that one or the other could become the dominating mechanism. It is plausible that in the rapidly responsive buds of decapitated pea, the first events are changes in sugar levels, while in Arabidopsis, this effect is less obvious. It is also possible that the sequence of events triggered by decapitation differs from the mechanisms involved in the slower bud activation conditions associated with developmental or nutritional changes (e.g., flowering or high P status).

## Competition Among Buds Kicks in *via* Auxin- and SL-Mediated Correlative Inhibition

Once a shoot is decapitated, numerous axillary meristems could potentially grow out. Even if not all of them are activated at the same time and with the same dynamics (depending, e.g., on their distance from the shoot tip), still several axillary buds may simultaneously be activated to grow. Hence, decapitation could potentially lead to bushy shoot phenotypes as in mutants with decreased AD (*max*, *dad*, *rms,* and *dwarf*). However, this is normally not the case, because the remaining buds are in mutual competition (Crawford et al., 2010; Shinohara et al., 2013; Balla et al., 2016; Paterlini et al., 2021), and often, one bud rapidly outcompetes all the others. Therefore, soon after decapitation, AD is reestablished resulting in a single new main shoot. It is plausible that this phenomenon is due to the rapid re-activation of correlative inhibition among the buds as a result of dominating auxin canalization in the new main shoot (Crawford et al., 2010; Shinohara et al., 2013; Balla et al., 2016; Paterlini et al., 2021). Hence, the SL-modulated auxin-based competition mechanism in AD is not only required to maintain axillary meristems in a silent state during normal development but also to quickly re-establish branching hierarchy after a disturbance (Domagalska and Leyser, 2011).

## Activation of the Cell Cycle and Establishment of a Symplastic Conduit for Resource Supply: A Paradigm for the Evolutionary Origin of Bud Activation Mechanisms?

Bud dormancy is a common phenomenon in perennial plants that have to cope with periods of harsh environmental conditions (e.g., cold winters; Rohde and Bhalerao, 2007). Dormancy and bud induction have been studied particularly well in birch, hybrid aspen, and various fruit trees (Arora et al., 2003). The notion that, in annual plants, the activation of axillary buds upon decapitation involves inductive signals in addition to the release from AD, is paralleled by studies on bud activation in perennials (Rohde and Bhalerao, 2007). After a dormant phase during winter, such plants activate their meristems (including the most apical dormant buds) in spring (Arora et al., 2003). Although not directly comparable, the hypothesis that bud activation in annual plants, and the induction of the winter buds in perennials, may share common elements of regulation, has received substantial support (Rohde and Bhalerao, 2007).

The meristems of perennials in an inactive state during the winter period are comparable to silent axillary buds of annuals with strong AD. In both cases, the cell cycle is nearly arrested, and symplastic connectivity appears to be reduced, involving the accumulation of callose in the phloem and in plasmodesmata (Tylewicz et al., 2018). Notably, similar regulatory circuits are involved in bud dormancy of annuals and perennials, including ABA and BRC1 (Liu and Sherif, 2019; Maurya et al., 2020; Azeez et al., 2021; Pan et al., 2021). In addition, bud dormancy and sprouting of potato tubers appears to involve mechanisms related to AD in annuals (Sonnewald and Sonnewald, 2014). Hence, the regulation of bud dormancy and bud activation in annuals and perennials may involve a shared mechanism with a common evolutionary origin.

Interestingly, ABA is also a central element in seed dormancy (Finkelstein et al., 2008), indicating that seed dormancy and bud dormancy could be regulated by similar hormonal pathways (Ruttink et al., 2007; Wang et al., 2016a). This analogy extends to the notion that, as in seed dormancy (Skubacz and Daszkowska-Golec, 2017; Tuan et al., 2018), bud outgrowth in perennials involves antagonistic interactions of ABA and gibberellic acid (GA; Pan et al., 2021). However, the role of GA is complex and context-dependent (Katyayini et al., 2020; Pan et al., 2021). Indeed, GA can promote (Rinne et al., 2011, 2016; Ni et al., 2015; Katyayini et al., 2020) or inhibit (Scott et al., 1967; Zheng et al., 2018; Katyayini et al., 2020) bud outgrowth, depending on the plant species, and on the developmental and environmental conditions, but in most cases, GA contributes to bud activation (Liu and Sherif, 2019; Pan et al., 2021).

## Conclusion

SL is produced in most parts of the plant, presumably along the vasculature, and it is mobile in an acropetal fashion by a mechanism that may involve the transpiration stream. The function of SL in AD acts at different levels of the plant. Attenuation of auxin transport capacity mediates bud dormancy by interfering with auxin canalization from the buds. In addition, SL can directly exert bud dormancy by inducing *BRC1*/*TB1*, and through the induction of ABA signaling. Bud release involves both inductive signals such as sucrose, T6P, and cytokinin, as well as the release from the inhibitory BRC1/TB1 and ABA. Ultimately, this results in the activation of the cell cycle and metabolism in the buds. Common patterns in the regulation of dormancy in axillary buds of annual plants, and in bud dormancy in perennials, suggest that the phenomena may be related.

## Author Contributions

DR, KK, and FS discussed and wrote the manuscript. All authors contributed to the article and approved the submitted version.

## Funding

The work has been funded by two grants from the Swiss National Science Foundation to DR (IZCSZ0 174608, 310030_200367), the COST action FA1206 (STREAM), and the “National Overseas Scholarship” to KK from the Indian Government.

## Conflict of Interest

The authors declare that the research was conducted in the absence of any commercial or financial relationships that could be construed as a potential conflict of interest.

## Publisher’s Note

All claims expressed in this article are solely those of the authors and do not necessarily represent those of their affiliated organizations, or those of the publisher, the editors and the reviewers. Any product that may be evaluated in this article, or claim that may be made by its manufacturer, is not guaranteed or endorsed by the publisher.

## Abbreviations

SL: Strigolactone
AD: Apical dominance
ABA: Abscisic acid
BRC1: BRANCHED1
CK: Cytokinin
P: Phosphorus
N: Nitrogen
GA: Gibberellic acid
